# Characterization of Imidazopyridine Compounds as Negative Allosteric Modulators of Proton-Sensing GPR4 in Extracellular Acidification-Induced Responses

**DOI:** 10.1371/journal.pone.0129334

**Published:** 2015-06-12

**Authors:** Ayaka Tobo, Masayuki Tobo, Takashi Nakakura, Masashi Ebara, Hideaki Tomura, Chihiro Mogi, Dong-Soon Im, Naoya Murata, Atsushi Kuwabara, Saki Ito, Hayato Fukuda, Mitsuhiro Arisawa, Satoshi Shuto, Michio Nakaya, Hitoshi Kurose, Koichi Sato, Fumikazu Okajima

**Affiliations:** 1 Laboratory of Signal Transduction, Institute for Molecular and Cellular Regulation, Gunma University, Maebashi, Japan; 2 Laboratory of Pharmacology, College of Pharmacy, Pusan National University, Busan, Republic of Korea; 3 Faculty of Pharmaceutical Science, Hokkaido University, Sapporo, Japan; 4 Center for Research and Education on Drug Discovery, Hokkaido University, Sapporo, Japan; 5 Department of Pharmacology and Toxicology, Graduate School of Pharmaceutical Sciences, Kyushu University, Fukuoka, Japan; Rutgers University, UNITED STATES

## Abstract

G protein-coupled receptor 4 (GPR4), previously proposed as the receptor for sphingosylphosphorylcholine, has recently been identified as the proton-sensing G protein-coupled receptor (GPCR) coupling to multiple intracellular signaling pathways, including the G_s_ protein/cAMP and G_13_ protein/Rho. In the present study, we characterized some imidazopyridine compounds as GPR4 modulators that modify GPR4 receptor function. In the cells that express proton-sensing GPCRs, including GPR4, OGR1, TDAG8, and G2A, extracellular acidification stimulates serum responsive element (SRE)-driven transcriptional activity, which has been shown to reflect Rho activity, with different proton sensitivities. Imidazopyridine compounds inhibited the moderately acidic pH-induced SRE activity only in GPR4-expressing cells. Acidic pH-stimulated cAMP accumulation, mRNA expression of inflammatory genes, and GPR4 internalization within GPR4-expressing cells were all inhibited by the GPR4 modulator. We further compared the inhibition property of the imidazopyridine compound with psychosine, which has been shown to selectively inhibit actions induced by proton-sensing GPCRs, including GPR4. In the GPR4 mutant, in which certain histidine residues were mutated to phenylalanine, proton sensitivity was significantly shifted to the right, and psychosine failed to further inhibit acidic pH-induced SRE activation. On the other hand, the imidazopyridine compound almost completely inhibited acidic pH-induced action in mutant GPR4. We conclude that some imidazopyridine compounds show specificity to GPR4 as negative allosteric modulators with a different action mode from psychosine, an antagonist susceptible to histidine residues, and are useful for characterizing GPR4-mediated acidic pH-induced biological actions.

## Introduction

OGR1-family G protein-coupled receptors (GPCRs), including ovarian cancer G protein-coupled receptor 1 (OGR1 or GPR68), G protein-coupled receptor 4 (GPR4), T-cell death-associated gene 8 (TDAG8 or GPR65), and G2A, have initially been reported as receptors for lysolipids, such as sphingosylphosphorylcholine (SPC) and lysophosphatidylcholine (LPC) [1–3]; however, lipid actions have not always been confirmed [4, 5]. Ludwig et al. reported that OGR1 and GPR4 sense extracellular pH, resulting in the activation of the phospholipase C/Ca^2+^ and adenylyl cyclase/cAMP signaling pathways through G_q/11_ and G_s_ proteins, respectively [4]. Later, proton sensitivity was also reported for TDAG8 [6]. Protonation of histidine residues on the extracellular domains of receptors has been suggested to cause conformational changes in the receptors, thereby facilitating the coupling with G proteins [4, 6, 7]. As for G2A, although proton sensitivity was detected, the receptor is constitutively active even at a neutral or alkaline pH [8]. Thus, it is controversial whether G2A senses changes in the extracellular pH in native cells that endogenously express G2A [9–11].

Extracellular acidification occurs at site of ischemia and inflammation [2, 12]. Recent studies have shown that OGR1-family GPCRs sense a change in extracellular pH and regulate cellular functions in a variety of cell types, including inflammatory cells under physiological pH and pathologically severe pH circumstances [5, 13, 14]. For example, OGR1 is involved in cyclooxygenase (COX)-2 expression in osteoblasts [15], prostaglandin production in vascular smooth muscle cells [16, 17], and interleukin-6 and connective tissue growth factor expression in airway smooth muscle cells [18, 19]. OGR1 has also been shown to be involved in airway inflammation *in vivo* [14, 20]. As for GPR4, the acidic pH has been shown to stimulate monocyte adhesion and expression of VCAM-1 and ICAM-1, in association with cAMP accumulation [21]. Moreover, GPR4 is suggested to be involved in acidic pH-induced expression of a number of inflammatory genes, including chemokines, cytokines, NF-κB pathway genes, COX-2, and stress response genes [22]. Therefore, the OGR1-family receptors may be potential targets for inflammatory diseases. The physiological and pathophysiological roles of OGR1-family GPCRs have been mainly characterized using knockdown cells and knockout mice. Only a few chemicals have been available for the characterization of proton-sensing GPCRs [5]. Chemicals that specifically affect GPR4 and OGR1 may be expected to be useful for treatment of inflammatory disorders, such as atherosclerosis and cancers. Some compounds that affect GPR4 activity have appeared in patent claims [23, 24]; however, no data were provided for their specificity.

In the present study, we characterized some imidazopyridine compounds that are described as inhibiting GPR4-mediated actions in the patent claim [23], and compared them with psychosine, a selective proton-sensing GPCR antagonist [6]. We found that these compounds are specific to GPR4. Thus, the chemicals inhibited the responses mediated by GPR4 but not those by OGR1, TDAG8, and G2A. We also found that the imidazopyridine compound can be applied to characterize the GPR4-mediated biological functions induced by extracellular acidification, *i*.*e*., inflammatory gene expression and receptor internalization.

## Materials and Methods

### Materials

Imidazopyridine compounds as GPR4 modulators; *i*.*e*., 2-((2-ethyl-5,7-dimethyl-3*H*-imidazo[4,5-*b*]pyridin-3-yl)methyl)-8-((4-methylpiperazin-1-yl)methyl)-10,11-dihydro-5*H*-dibenzo[*b*,*f*]azepine fumaric acid salt (compound 1), 4-((2-ethyl-5,7-dimethyl-3*H*-imidazo[4,5-*b*]pyridin-3-yl)methyl)-*N*-((1*s*,4*s*)-4-(4-methylpiperazin-1-yl)methyl)cyclohexyl)aniline (compound 2), and 2-((2-ethyl-5,7-dimethyl-3*H*-imidazo[4,5-*b*]pyridin-3-yl)methyl) -10,11-dihydro-5*H*-dibenzo[*b*,*f*]azepine (compound 3) were synthesized by us according to the methods previously described in the patent [23]. Purities of these compounds were confirmed by elemental analysis or HPLC analysis. Compound 1: Elemental analysis calculated for C_39_H_46_N_6_O_8_·0.5H_2_O: C, 63.66; H, 6.44; N, 11.42. Found: C, 63.64; H, 6.24; N, 11.26. Compound 2: 97.7% HPLC purity (column: YMC-pack SIL 4.6 x 150 mm, eluent: CHCl_3_: MeOH: triethylamine = 60: 40: 0.02, 1.0 ml/min, 20°C, 260 nm; retention time 4.0 min. Compound 3: Elemental analysis calculated for C_25_H_26_N_4_·0.7H_2_O: C, 76.00; H, 6.99; N, 14.18. Found: C, 75.96; H, 6.75; N, 13.89. Psychosine or galactosylsphingosine was purchased from Sigma-Aldrich (St Louis, MO); *N*-acetyl-psychosine was from Matreya LLC (Pleasant Gap, PA); fatty acid-free bovine serum albumin (BSA) was from Calbiochem-Novabiochem Co. (San Diego, CA); [Arg^8^]-vasopressin was Peptide Institute (Osaka, Japan); cyclic AMP EIA Kit was from Cayman Chemical Co. (Ann Arbor, MI); Fura-2/acetoxymethylester (Fura-2/AM) was from Dojindo (Tokyo, Japan); and Lipofectamine 2000 Reagent was from Invitrogen (Carlsbad, CA). RT-PCR probes specific for VCAM-1 (Hs01003372), ICAM-1 (Hs00164932), chemokine (C-X-C motif) ligand 2 (CXCL2, Hs00601975), inerleukin-8 (IL-8, Hs00174103), and glyceraldehydes 3-phosphate dehydrogenase (GAPDH, 4352934E) were from Applied Biosystems (Foster City, CA). HEK293 cells that express green fluorescent protein (GFP)-conjugated mouse vasopressin V_1a_ receptor [25] were generously gifted by Drs. Hirasawa and Tsujimoto of Kyoto University. The sources of all other reagents were the same as described previously [6, 7, 16, 17, 26].

### Preparation of receptor cDNA plasmids

The cDNAs for proton-sensing GPCR cDNAs, including TDAG8, G2A, OGR1, and GPR4 were amplified from a human cDNA library by RT-PCR as described previously [6, 7, 26]. To construct the TDAG8 and G2A receptor expression plasmids, the entire coding region of the TDAG8 (1014 bp, NM_003608) and the G2A (1142 bp, NM_013345) were subcloned into the EcoRI site of the pEFneo eukaryotic expression vector [6, 26], respectively. The entire coding region of OGR1 (1128 bp, NM_003485) was amplified by RT-PCR with the 5’-primer (aagcttccaccATGAGGAGTGTGGCCCCTTCAGGCCCAAAGATGGGGAACATCACTGCAGACAACTCC) and the 3’-primer (gaattcCTAGGCCAACCTGCCCGTGGGGAA). The OGR1 fragment was subcloned into HindIII/EcoRI sites of pcDNA3.1 (Life Technologies, Osaka, Japan). The HEK293 cells transiently transfected with the OGR1 construct showed proton concentration-dependent increases in SRE-driven transcriptional activity consistent with the previous results with OGR1 (1098 bp, NM_003485) in pEFneo [6, 26]. The amplified fragment containing GPR4 (1089 bp, NM_005282) was subcloned into HindIII/EcoRI sites of pcDNA3.1 [7]. The H79F mutant and H165F/H269F double mutant of GPR4, in which 79th and both 165th and 269th histidine residues from the N-terminus were changed to phenylalanine, were generated by PCR-based mutagenesis and also cloned into the Hind III/Eco RI site of pcDNA 3.1 [7]. To tag the C terminus of the receptors with GFP, the stop codon was removed and cloned into pEGFP-N2 (Life Technologies, Osaka, Japan), as described previously [7]. The amplified GPR4 fragment was also subcloned into EcoRI site of a pIRESneo expression vector and the GPR4 plasmid was used for the preparation of permanent cell line of Chinese hamster ovary (CHO) cells resistant to neomycin (G418 sulfate at 1 mg/ml) [6].

### Cell culture

HEK293 cells were cultured in Dulbecco’s modified Eagle’s medium (DMEM) containing 10% (v/v) fetal bovine serum (FBS) (Life Technologies, Osaka, Japan) and used for serum responsive element (SRE) promoter activity. Permanent cell line of CHO cells expressing GPR4 were cultured in DMEM containing 10% FBS for measurement of cAMP. COS7 cells were transiently transfected by electroporation with GPR4-pcDNA3.1 or TDAG8-pEFneo and cultured for 2 days in DMEM containing 10% FBS [6]. Human aortic smooth muscle cells (AoSMCs) were obtained from Kurabo Bio-Medical department (Osaka, Japan) and cultured as described previously [17]. Human umbilical vascular endothelial cells (HUVECs) (passage number 3) and HuMedia-EG2 (KE-2150S) were obtained from Kurabo Bio-Medical department (Oosaka, Japan). The cells were cultured in HuMedia-EG2 supplemented with 2% FBS and several growth factors as previously described [27]. All the cells were cultured in a humidified air/CO_2_ (19:1) atmosphere.

### Accumulation of cAMP in CHO cells

CHO cells and COS7 cells (2 x 10^5^ cells) were cultured on plated on 24-multiplates. Twenty-four hours before the experiments, the medium was changed to fresh DMEM (without serum) containing 0.1% fatty acid-free BSA. The cells were washed once and preincubated for 10 min at 37°C in the HEPES-buffered medium (pH 7.6). The HEPES-buffered medium consisted of 20 mM HEPES (pH 7.6), 134 mM NaCl, 4.7 mM KCl, 1.2 mM KH_2_PO_4_, 1.2 mM MgSO_4_, 2 mM CaCl_2_, 2.5 mM NaHCO_3_, 5 mM glucose, and 0.1% BSA. The cells were then incubated for 30 min under the indicated pH in the presence of 0.5 mM 3-isobutyl-1-methylxanthine (IBMX) in a final volume of 0.5 ml [6]. The medium pH was adjusted by adding HCl or NaOH. All data in this report are referenced to pH at room temperature. The reaction was terminated by adding 100 μl of 1 N HCl. Cyclic AMP in the acid extract was measured by Cyclic AMP EIA Kit, as described previously [28].

### Dual luciferase reporter assay

SRE-driven promoter activity was assayed using the PathDetect Signal Transduction Pathway cis-Reporting Systems (Stratagene, La Jolla, CA). The reporter construct or pSRE-luc has firefly luciferase gene under the control of DNA-binding elements of *fos* gene for SRE. HEK293 cells were transfected in suspension (about 10^6^ cells/ml) with pSRE-luc (50 ng/ml) and pRL-TK (Promega, Madison, WI; 10 ng/ml) together with the respective receptor-expression plasmid (GPR4, OGR1, TDAG8, G2A, or GPR4 mutant; 10 ng/ml, unless otherwise stated) by using Lipofectamine 2000 Reagent according to the instructions. The cells were then further cultured in 12-multiplates (1 ml/well) for 12 h in growth culture medium and for another 16 h in serum-starved DMEM medium containing 0.1% BSA. The medium was changed to 25 mM HEPES-buffered DMEM medium (without serum) containing 0.1% BSA with appropriate pH in the presence of test agents and the cells were then incubated for 6 h. The cells of each well were lysed in reporter lysis buffer (Promega, Madison, WI) and luciferase activity was assayed using Dual-Luciferase Reporter Assay System (Promega, Madison, WI). Firefly luciferase activity (pSRE-luc) in each well was normalized to the Renilla luciferase activity (pRL-TK). The ratio of firefly and Renilla luciferase activities was used as the indicator for transcriptional activation. For further details see the previous paper [7].

### Measurement of [Ca^2+^]_i_

Twenty-four hours before the experiments, AoSMC culture medium was changed to fresh DMEM without serum containing 0.1% BSA for measurement of [Ca^2+^]_i_. The cells on 10-cm dish were gently harvested from dishes with phosphate-buffered saline (PBS) containing 0.05% trypsin-EDTA. The cells were incubated with 1 μM Fura-2/AM and [Ca^2+^]_i_ was estimated from the changes in the intensities of 540 nm fluorescence obtained by the two excitations (340 nm and 380 nm), which were monitored by CAF-110 fluorometer (JASCO, Tokyo, Japan), as described previously [28].

### Infection of adenovirus GPR4 and measurement of mRNAs of inflammatory genes

The recombinant adenovirus for GPR4 was obtained from BioFocus (A Galapagos Company, Leiden, The Netherlands) and infected as described previously [29]. In brief, 80% confluent HUVECs were infected with recombinant at a multiplicity of infection of 100 for 2 h at 37°C in DMEM containing 10% FBS. Cells were then cultured for an additional 24 h with DMEM containing 10% FBS. Under these conditions, infection with adenovirus coding GFP resulted in almost 100% cells positive to GFP. The cells are serum starved for 6 h and then incubated for 5 h with 25 mM HEPES-buffered RPMI1640 containing 0.1%BSA. Total RNA was prepared from HUVECs according to the manufacturer’s instructions for RNAiso Plus (Takara Bio Inc., Otsu, Japan). Quantitative real-time PCR (RT-qPCR) was performed by TaqMan technology, as described previously [30]. The expression level of the target mRNA was normalized to the relative ratio (x 10^3^) of the expression of GAPDH mRNA. The RT-qPCR assay was performed with 3 different RNA concentrations in each sample.

### Analysis by confocal laser scanning microscope

HEK293 cells were transfected with GFP-conjugated GPR4 and a permanent cell line was selected with G418 sulfate at 1 mg/ml as described [6]. Permanent cell line expressing GFP-conjugated GPR4 and V_1a_-VR were grown in 12-mm cover glass (Matsunami Glass Ind., Ltd., Osaka, Japan). The cells were incubated with test agents at pH 7.4 or 6.6 for 1 h and then washed with PBS and fixed with 3.7% formaldehyde in PBS for 30 min at room temperature. The fixed cells were stained with 4’, 6-diamidino-2-phenylindole (DAPI) in PBS for 30 min at 37°C and mounted in Permafluor (Immunon, PA, USA) on slide glass. Analysis was performed at 405 nm and 488 nm laser line to excite DAPI and GFP, respectively, using a confocal laser scanning microscope (FV1000-D, Olympus, Tokyo, Japan).

### Data presentation

All experiments were performed in duplicate or triplicate. The results of multiple observations are presented as the mean ± SEM from more than three different batches of cells, unless otherwise stated. Statistical significance was assessed by the Student’s *t*-test; values were considered significant at *p* < 0.05.

## Results

### Specific inhibition by imidazopyridine compounds of GPR4-mediated SRE-driven transcriptional activation in response to extracellular acidification

**Fig 1:**
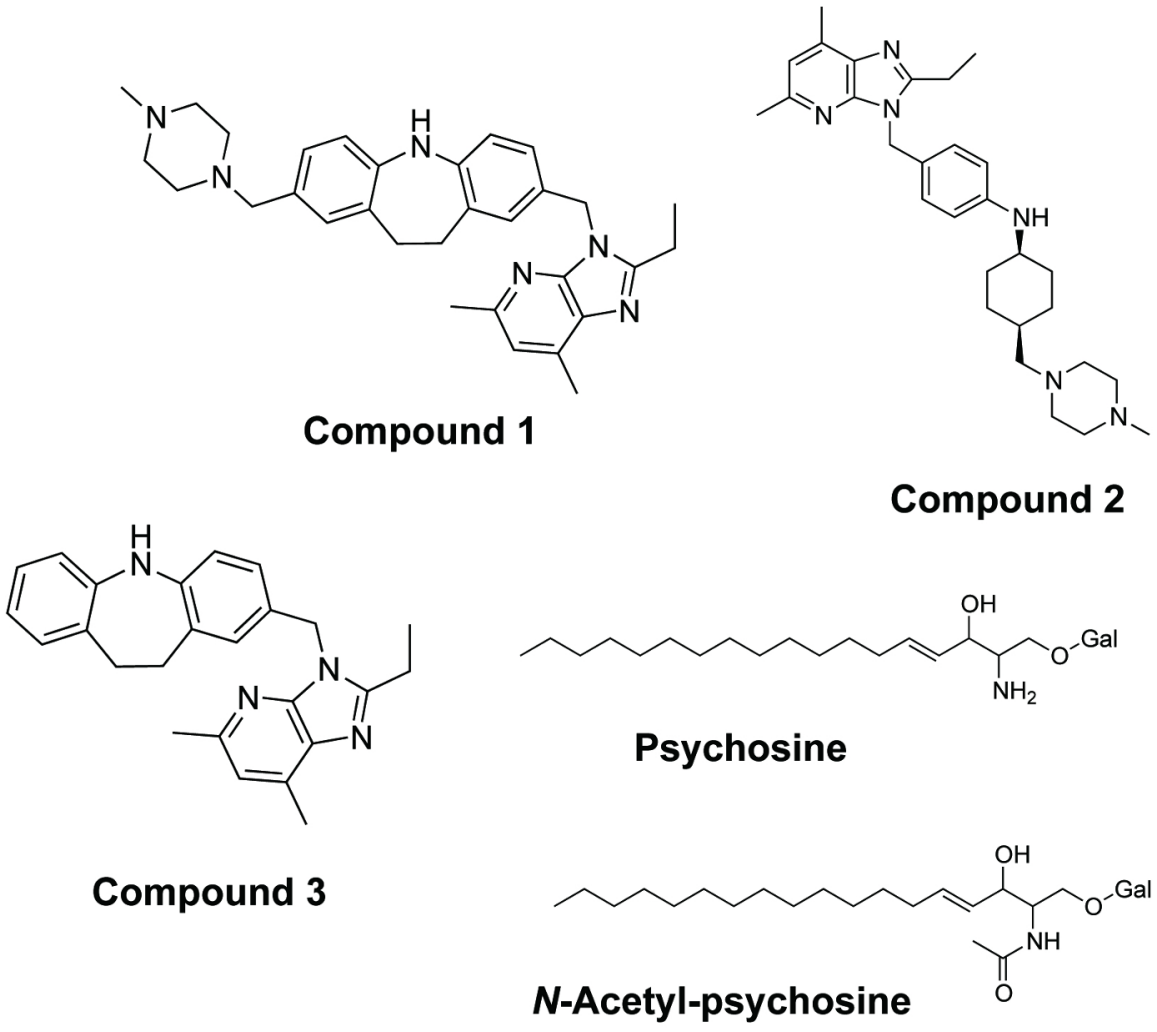
Chemical structure of imidazopyridine compounds, psychosine, and *N*-acetyl-psychosine. Chemical structures of imidazopyridine compounds, *i*.*e*., compound 1, 2, and 3, and psychosine (or galactosyl (Gal) sphingosine) and *N*-acetyl-psychosine are shown.

The chemical structures of imidazopyridine compounds that were characterized in the present study, *i*.*e*., compound 1, 2, and 3, are described in Fig 1. In this figure, the chemical structure of psychosine (galactosylsphingosine), which shows a selective inhibitory property for proton-sensing GPCRs, including GPR4, OGR1, and TDAG8 [6], and *N*-acetyl-psychosine was also shown.

**Fig 2:**
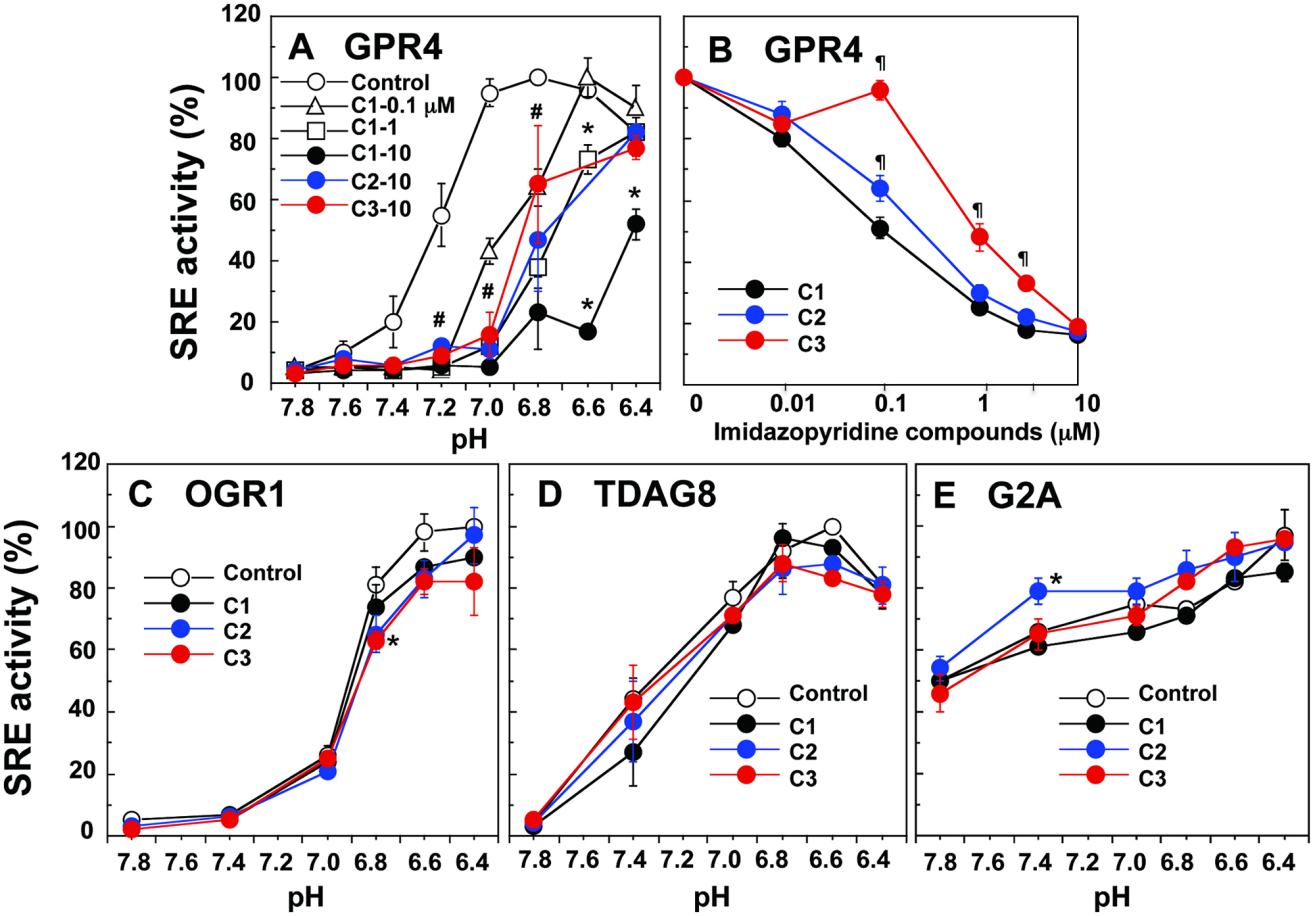
Specific inhibition of GPR4-mediated SRE promoter activity by imidazopyridine compounds. HEK293 cells were transiently transfected with GPR4 (A and B), OGR1 (C), TDAG8 (D), or G2A (E) plasmid, together with pRL-TK and pSRE-luc. The cells expressing the respective receptor and luciferase genes were then incubated for 6 h at the indicated pH to measure SRE promoter activity in the presence or absence of test compounds. The concentration of test compounds was as follows; 0.1 to 10 μM for compound 1 (C1) and 10 μM for compound 2 (C2) and compound 3 (C3) in (A), the indicated concentration in (B), and 10 μM for all the test compounds in (C to E). SRE activity was estimated as the ratio of firefly (pSRE-luc) and Renilla (pRL-TK) luciferase activities and the results are shown as percentages of the maximal activity (ratio) obtained at optimum pH for each receptor in (A and C-E): the maximal activity was 12.1 ± 3.8 for GPR4 at pH 6.8 in (A), 21.4 ± 7.1 for OGR1 at pH 6.4 in (C), 36.4 ± 0.4 for TDAG8 at pH 6.6 in (D), and 18.6 ± 6.7 for G2A at pH 6.4 in (E). In (B), the activity was expressed as percentages of the value obtained at pH 7.0. Results are means ± SEM of 4 to 6 determinations from two to three separate experiments. In (A), effect of each test compound is significantly different from control (*p < 0.05), while effects of all the test compounds at pH 7.2, 7.0, and 6.8 are significantly different from control (#*p* < 0.05). In (B), effect of C2 or C3 is significantly different from that of C1 (¶*p* < 0.05). In (C to E), effect of each test compound is significantly different from control (*p < 0.05).

HEK293 cells were transiently transfected with pcDNA 3.1 vector or wild-type GPR4 constructs. Unless otherwise stated, GPR4 refers to wild-type GPR4. Consistent with the previous results [26], GPR4-transfected cells showed proton concentration-dependent increases in SRE-driven transcriptional activity (Fig 2A). Activation of the SRE promoter has been shown to be mediated by G_13_ proteins/Rho signaling pathways [26]. Activation was biphasic: with decreasing extracellular pH, activity increased to a maximum at approximately pH 7.0 or 6.8 and then gradually decreased at a pH lower than 6.6. We synthesized several imidazopyridine compounds and screened for GPR4 negative modulator activity. Among them, we characterized in detail the specificity and affinity of three compounds. In the presence of appropriate concentrations of either compound 1, 2, or 3, the proton-dependent SRE responses were modulated with different inhibitory patterns that depended on the chemicals and their concentrations in GPR4-expressing HEK293 cells (Fig 2A). Compound 1 is the most effective among them, and 1 μM compound 1 was roughly comparable to 10-times higher concentrations of compound 2 or 3 (Fig 2A). To further compare their potency, the effects of increasing concentrations of these compounds on pH 7.0-induced SRE activation were examined (Fig 2B). Consistent with the results of Fig 2A, the order of potency for inhibiting proton-induced SRE activation was compound 1>2>3 (Fig 2B).

Extracellular acidification also stimulated SRE activity in HEK293 cells that expressed either OGR1 or TDAG8, although their proton sensitivity was slightly different from that of GPR4 (Fig 2C and 2D). In the case of G2A, although the basal activity observed at pH 7.8 was very high and proton sensitivity was not so high as other proton-sensing GPCRs, acidic pH slightly but significantly stimulates SRE activity (Fig 2E). Imidazopyridine compounds, however, failed to appreciably inhibit acidic pH-induced SRE activity in any case, although compounds 2 and 3 exerted small but significant effects in a few points (Fig2C–2E). Currently, however, whether the effects reflect the weak specificity of these compounds or simply reflect the small number of experiments is not known.

### Application of the GPR4 modulators for the characterization of the receptor-specific biological actions

**Fig 3:**
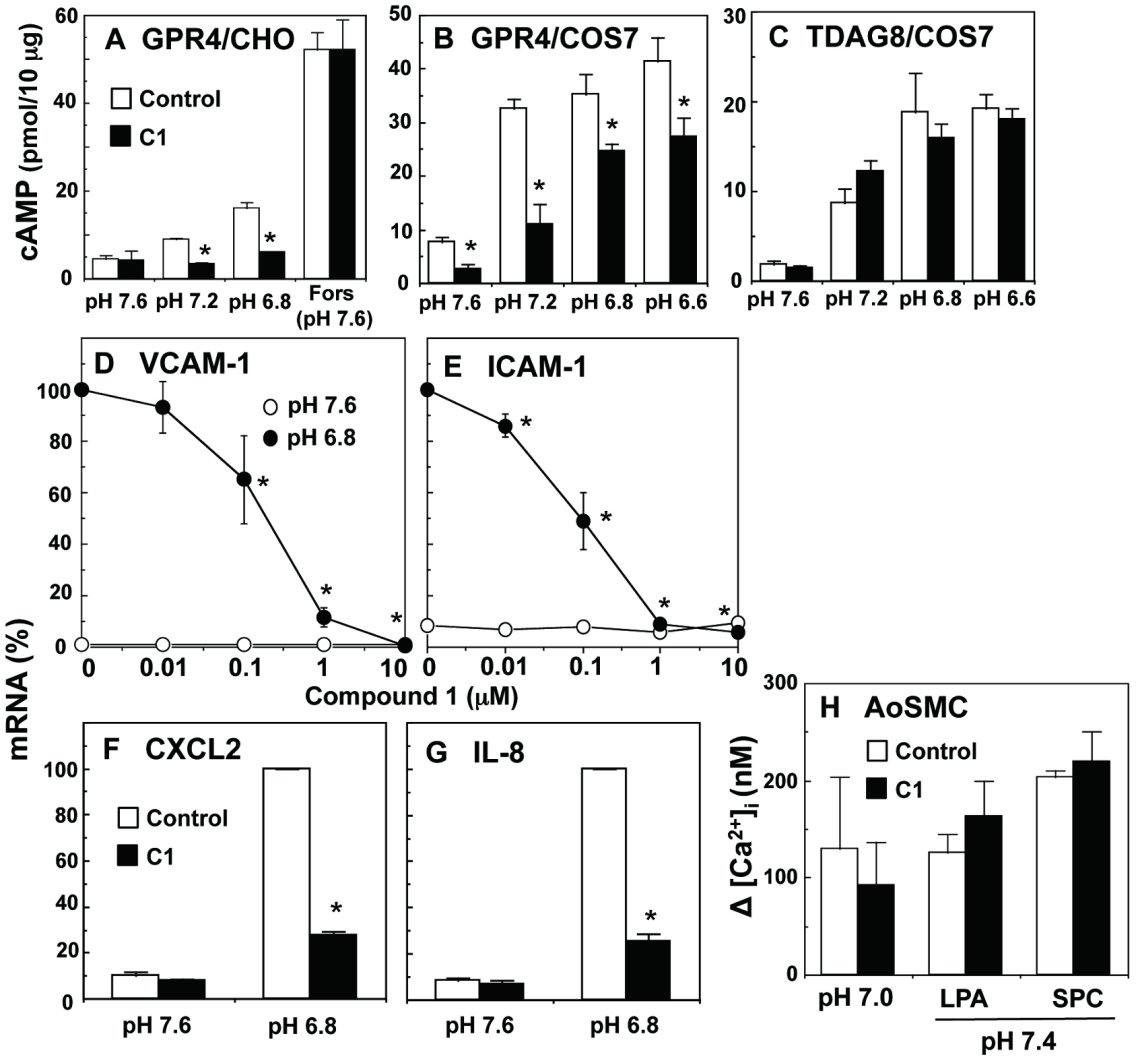
Compound 1 specifically inhibits GPR4-mediated responses. GPR4-expressing CHO cells (A), GPR4-expressing COS7 cells (B), or TDAG8-expressing COS7 cells (C) were incubated for 30 min to measure cAMP accumulation under the indicated pH with or without 1 μM compound 1 (C1) and/or 1 μM forskolin (Fors). Results are expressed as means ± SD of three determinations of the representative experiment in (A) and expressed as means ± SEM of five determinations of three separate experiments in (B and C). The effect of compound 1 was significant (*p < 0.05). HUVECs infected with GPR4 adenovirus were incubated for 6 h in the presence of the indicated concentrations of compound 1 in (D and E) or in the presence of absence of 1 μM compound 1 in (F and G) at the indicated pH to measure mRNAs for VCAM-1 (D), ICAM-1 (E), CXCL2 (F), and IL-8 (G). The mRNA expression normalized to GAPDH was expressed as percentages of the value in the absence of compound 1 at pH 6.8. These values at pH 6.8 were 22.7 ± 6.6 for VCAM1 mRNA, 15.1 ± 4.1 for ICAM1 mRNA, 226 ± 20 for CXCL2 mRNA, 12.9 ± 0.9 for IL-8 mRNA (normalized to GAPDH x 10^3^). The results are means ± SEM of three to five separate experiments. The effect of compound 1 was significant (*p < 0.05). (H) AoSMCs harvested from 10-cm dish were prelabeled with fura-2/AM. The cells were first incubated with compound 1 (1 μM) and then further incubated under indicated pH with or without LPA (1 μM) and SPC (20 μM) to monitor [Ca^2+^]_i_. The net [Ca^2+^]_i_ change (peak value-basal value) at around 15 s was calculated. Data are means ± SEM from three separate experiments.

Henceforth, we will focus on compound 1 as a GPR4 negative modulator. It is well known that GPR4 is coupled to cAMP signaling pathways [4, 26] in addition to Rho signaling pathways [26]. Consistent with previous results [6], CHO cells expressing GPR4 exhibited cAMP accumulation in response to extracellular acidification and the cAMP response was remarkably inhibited by compound 1, whereas the GPR4 modulator was ineffective for nucleotide accumulation induced by forskolin, an adenylyl cyclase activator (Fig 3A). Moreover, compound 1 inhibited acidic pH-induced cAMP accumulation in COS7 cells expressing GPR4 (Fig 3B) but not TDAG8 (Fig 3C). In COS7 cells, cAMP accumulation at basal condition of pH 7.6 was inhibited by compound 1 in GPR4-expressing cells but not TDAG8-expressing cells, suggesting that GPR4 is activated even by 25 nM protons present at pH 7.6 when expressed in COS7 cells. A recent study showed that GPR4 activation leads to the expression of inflammatory genes such as adhesion molecules, chemokines, and cytokines through cAMP signaling pathways [21, 22]. We examined the effect of compound 1 on the acidic pH-induced expression of mRNAs of these inflammatory genes in GPR4-expressing HUVECs. As shown in Fig 3D to 3G, the GPR4 modulator inhibited acidic pH-induced mRNA expression of VCAM-1, ICAM-1, CXCL2, and IL-8.

We have previously shown that the Ca^2+^ response to acidification is mediated by OGR1/G_q/11_ proteins in AoSMCs [16]. As shown in Fig 3H, the Ca^2+^ response to pH 7.0 was not appreciably affected by compound 1. Similarly, the Ca^2+^ response to lysophosphatidic acid (LPA) and SPC, both of which may also be mediated through G_q/11_ proteins [16, 17], was hardly affected by the GPR4 modulator. Thus, compound 1 specifically inhibited GPR4/G_s_ protein-mediated actions but not TDAG8/G_s_ protein- and OGR1/G_q/11_ protein-mediated actions in cell types other than HEK293 cells.

**Fig 4:**
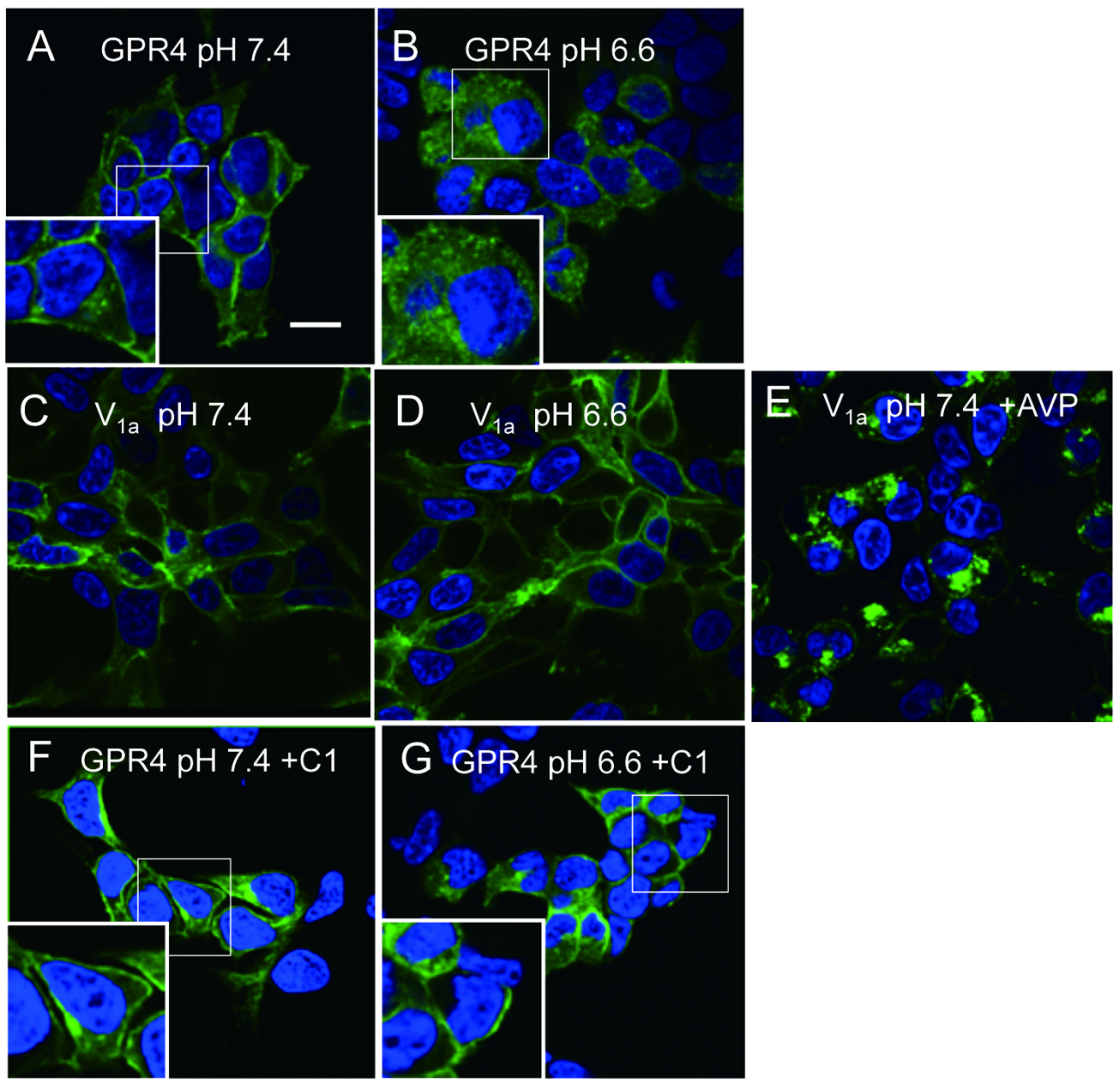
Internalization of GPR4 in response to acidification and its inhibition by compound 1. Permanent cell line expressing GFP-conjugated GPR4 or V_1a_ vasopressin receptor was incubated for 1 h with or without 1 μM [Arg^8^]-vasopressin (AVP) or 1 μM compound 1 (C1) under the indicated pH to monitor of receptor internalization. After fixation, nucleus was stained with DAPI (blue) and localization of GFP-receptor (green) was monitored. Scale bar: 20 μm. The results are representative of three to four separate experiments. The higher magnification image of the small square is shown in the large square.

Proton is a unique ligand for the activation of GPCRs: it may activate the receptor through the protonation of certain extracellular histidine residues [4, 7], whereas most GPCR ligands may interact with a specific region of a receptor. Ligand activation of GPCRs has been shown to cause redistribution of the receptors from the cell surface to the intracellular space through a process of endocytosis known as internalization [31]. It is interesting to consider whether or not GPR4 is internalized in response to acidic pH and whether the GPR4 modulator attenuates the receptor processing. For this purpose, GFP-tagged GPR4 was monitored. Using a confocal laser scanning fluorescence microscope, we acquired fluorescent images of cells that expressed GFP-labeled GPR4 (Fig 4). At pH 7.4, GFP-GPR4 was extensively detected on the plasma membrane (Fig 4A). Under the acidic pH of 6.6 for 1 h, however, a clear GFP localization in the plasma membrane was lost with a concomitant increase in fluorescent signals inside the cells (Fig 4B). The acidic pH effect was a specific action. Thus, the plasma membrane GFP-vasopressin V_1a_ receptor at pH 7.4 (Fig 4C) was still localized in the plasma membrane by the acidic pH of 6.6 (Fig 4D), whereas [Arg^8^]-vasopressin effectively facilitated internalization of the receptor to inside the cells at pH 7.4 (Fig 4E). Finally, we evaluated the effect of compound 1. The clear fluorescent intensity was visualized in a plasma membrane either at pH 7.4 (Fig 4F) or at pH 6.6 (Fig 4G) in the presence of the compound 1. Thus, compound 1 inhibited acidic pH-induced internalization of GPR4.

### Comparison of action modes of imidazopyridine compounds and psychosine, a selective antagonist for proton-sensing GPCRs

**Fig 5:**
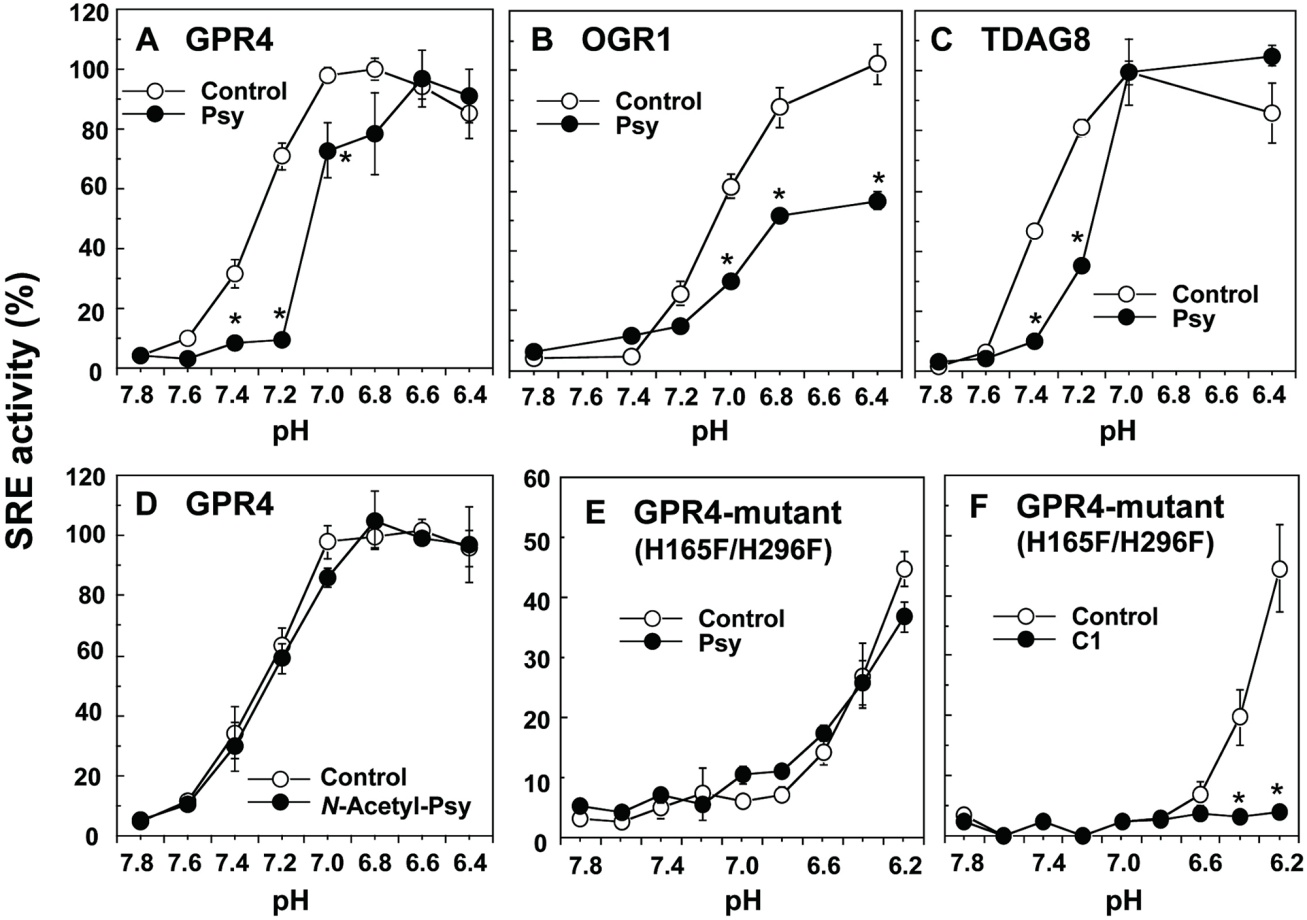
Different action modes of psychosine from compound 1. HEK293 cells were transiently transfected with plasmid of GPR4 (A and D), OGR1 (B), TDAG8 (C), or H165F/H269F double mutant of GPR4 (GPR4-mutant; E and F), together with pRL-TK and pSRE-luc. The cells expressing the respective receptor and luciferase genes were then incubated for 6 h at the indicated pH to measure SRE promoter activity in the presence or absence of psychosine (Psy; 10 μM), *N*-acetyl-psychosine (*N*Ac-Psy; 10 μM), or compound 1 (C1; 100 nM). SRE activity was estimated as the ratio of firefly (pSRE-luc) and Renilla (pRL-TK) luciferase activities and the results are shown as percentages of the maximal activity (ratio) obtained at optimum pH for each receptor in (A-D), as shown in Fig 2. In GPR4 mutant experiments (E and F), the activity was expressed as percentages of the value obtained at pH 6.8 in wild-type GPR4 (A). Results are means ± SEM of 4 to 6 determinations from two to three separate experiments. The effect of each test compound was significantly different from control (*p < 0.05).

Psychosine, or galactosylsphingosine, has been known to accumulate in Krabbe’s disease, a hereditary metabolic disorder, and results in apoptosis of oligodendrocytes, inhibition of cytokinesis, and globoid cell formation [32–35]. However, the mechanism by which psychosine induces these toxic effects is not fully understood [35]. We have previously shown that the lysolipid inhibited the acidification-induced TDAG8- and GPR4-mediated cAMP accumulation and OGR1-mediated inositol phosphate production, whereas it did not appreciably inhibit similar responses to other GPCR agonists [6, 36]. Thus, psychosine selectively inhibited proton-sensing GPCR-mediated actions, although only a narrow range of concentrations was effectively applied [6], and the specificity was not always been observed [3]. Consistent with our previous results [6], psychosine inhibited acidification-induced SRE activation in GPR4 (Fig 5A), OGR1 (Fig 5B), and TDAG8 (Fig 5C). A non-selective or detergent action of psychosine can be ruled out. Thus, *N*-acetyl-psychosine, in which the primary amino group of psychosine is acetylated (Fig 1), was ineffective for proton-induced SRE activation (Fig 5D). The result also suggests that the primary amino group of psychosine is involved in its antagonistic action.

We have previously shown that histidine residues at 79th, 165th, and 269th from the N-terminus are responsible for proton sensing [7]. The role of histidine residues in inhibitory psychosine action was further examined by using H79F mutant GPR4 and H165F/H296F double mutant GPR4. When H79F mutant was used, maximal activity was shifted to the right from at around pH 7.0 to pH 6.6 without change in the maximum (compare Fig 5A and S1 Fig); however, either 10 μM psychosine or 100 nM compound 1, whose inhibitory action was comparable each other (Fig 2A and Fig 5A), inhibited acidic pH-induced SRE activation (S1 Fig). These results suggest that H79 has a minor role for the inhibition by either psychosine and compound 1. On the other hand, as for the H165F/H296F double mutant GPR4, the maximal activity was roughly 50% of that of wild-type GPR4 at pH 6.2, the lowest pH examined in the present study, in the mutant GPR4 (Fig 5E and 5F). Interestingly, in the mutant GPR4-expressing cells, psychosine was ineffective for the further inhibition of acidic pH-induced action (Fig 5E). On the other hand, compound 1 almost completely inhibited action induced by any proton concentration employed (Fig 5F). These results suggest that action modes of psychosine and the GPR4 modulator are different with respect to their susceptibility to proton-sensing histidine residues in GPR4.

## Discussion

**Fig 6:**
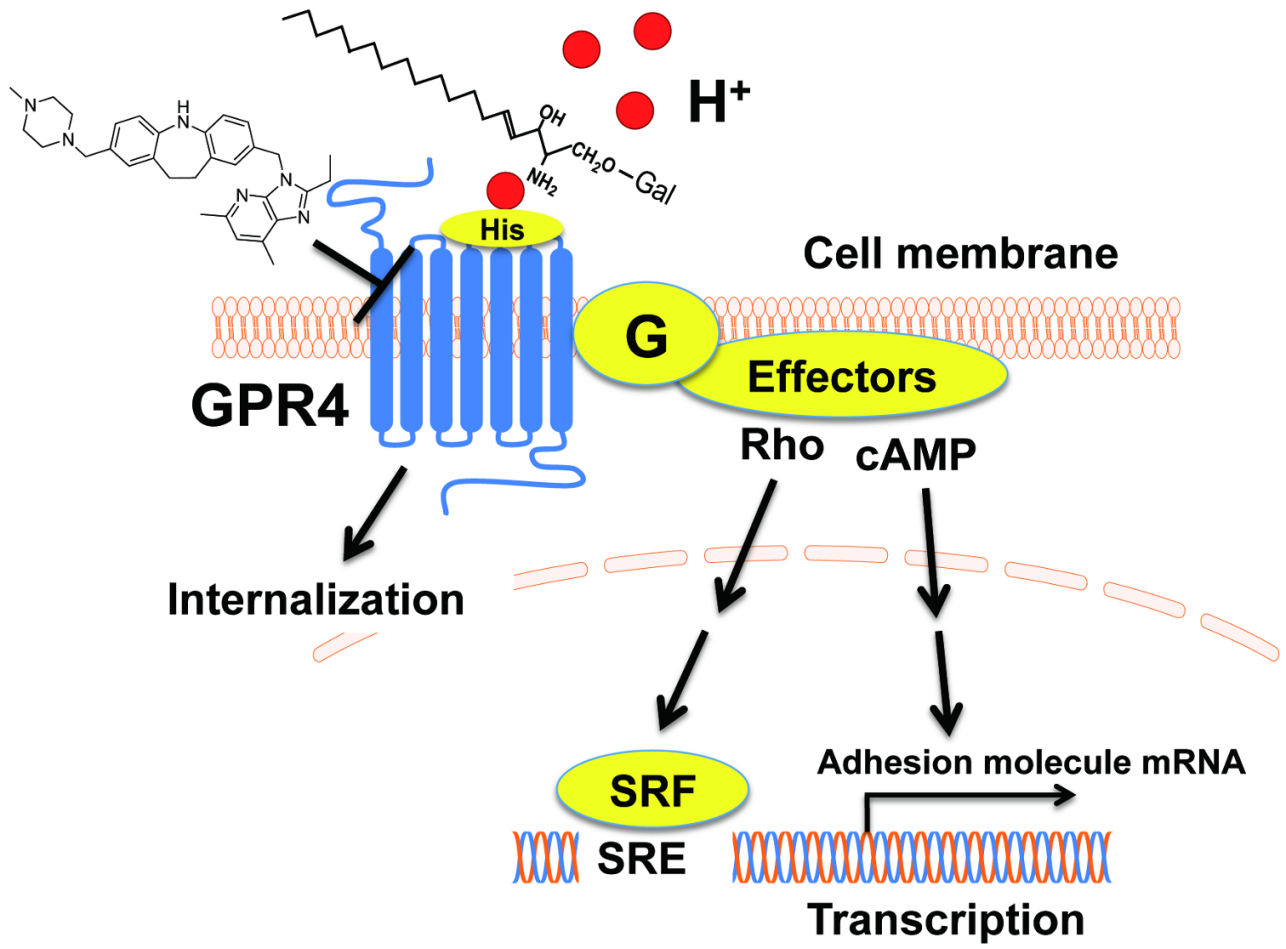
GPR4 signaling pathways and action modes of the imidazopyridine compound and psychosine as the GPR4 modulator and antagonist. Extracellular protons induce GPR4 activation through histidine residues and subsequent activation of G protein/effector systems, *i*.*e*., G_s_/cAMP system and G_13_/Rho system, resulting in the mRNA expression of adhesion molecules and SRE transcriptional activation, respectively. Similarly to other GPCRs, GPR4 is desensitized by its internalization in response to extracellular acidification. Both imidazopyridine compound and psychosine inhibit the proton/GPR4 function but by different action modes with respect to the histidine susceptibility. See text more detail.

In the present study, we have shown that some imidazopyridine compounds specifically and negatively modulate GPR4-mediated actions in response to acidic pH without appreciable effects on OGR1- and TDAG8-mediated actions. We also compared the action modes of imidazopyridine compounds with those of psychosine, a causal lysolipid in a hereditary metabolic disorder or Krabbe’s disease, which has been shown to selectively inhibit proton-sensing GPCR-mediated actions. We found that imidazopyridine compounds are not as susceptible as psychosine to histidine residues, which are critically involved in psychosine-induced inhibition of GPR4 activity. The postulated GPR4 signaling pathways and action modes of imidazopyridine compounds and psychosine are shown in Fig 6.

SRE activation in response to acidic pH in cells that express proton-sensing GPCRs, including GPR4, OGR1, and TDAG8, has been shown to be mediated by common signaling pathways involving G_13_ proteins and Rho, as evidenced by the finding that acidic pH-induced SRE activation was inhibited by siRNA specific to Gα_13_, p115 regulator of G protein signaling (p115RGS) (a dominant negative p115RhoGEF), and C3 toxin (a specific inhibitor of Rho through ADP-ribosylation) [26]. The GPR4 modulator specifically inhibited the GPR4/G_s_ protein-mediated but not TDAG8/G_s_ protein-mediated cAMP response to acidic pH without change in the response to forskolin. We also observed that the GPR4 modulator failed to affect LPA- and SPC-induced Ca^2+^ mobilization, a characteristic G_q/11_ protein-mediated response, in human smooth muscle cells. These results suggest that imidazopyridine compounds may modulate GPR4 receptor itself without affecting G proteins and downstream signaling pathways.

The action mode of the GPR4 modulator, however, may be different from that of psychosine, which has been reported to be a selective antagonist for proton-sensing GPCRs, including GPR4 [6]. The lysolipid has first been suggested to be an agonist for TDAG8, which was assumed to be coupled to inhibitory cAMP signaling, based on the inhibition of forskolin-induced cAMP accumulation [37]. The reason of the discrepancy of the conclusion between the latter study [37] and ours [6] is not known. However, we tentatively speculate that TDAG8-mediated cAMP accumulation induced by protons in medium might be inhibited by psychosine in that study. Proton-sensing GPCRs may sense extracellular protons through histidine residues on the receptor proteins. Thus, double mutation of H165 and H269 to phenylalanine in GPR4 shifted the optimum pH from around 7.0 in wild-type GPR4 to a more acidic pH of 6.2 or less. These results suggest that histidine residues are orthosteric sites of GPR4 [38]. Interestingly, psychosine was ineffective for acidic pH-induced SRE activation in mutant GPR4 (H165F/H269F)-expressing cells, in which critical orthosteric sites of GPR4 are lacking, whereas the GPR4 modulator almost completely inhibited psychosine-insensitive acidic pH-induced action in the same cells. These results suggest that psychosine interferes with GPR4 activation, possibly by inhibiting protonation of histidine residues through a primary amino group of lysolipids. Indeed, *N*-acetyl-psychosine was ineffective for inhibiting GPR4-mediated action. On the other hand, imidazopyridine compounds that have no primary amino group in molecules (Fig 1), may affect a certain receptor region that specifies GPR4 independent of histidine residues (Fig 6), although the precise action modes of psychosine and GPR4 modulators need further characterization. We tentatively speculate that psychosine behaves as an orthosteric antagonist and that the imidazopyridine compound behaves as a negative allosteric modulator [38] of GPR4. As an example of the negative allosteric modulator, a recent study has shown that boronic acid derivatives for chemokine receptor CXCR3 act on second allosteric binding pocket of the receptor to modulate receptor functions [39].

GPCRs bind to a diverse array of ligands, including amines, peptides, and lipids, which consequently induces conformational changes in the receptor and dissociation of G proteins into an α subunit and βγ subunits and activates downstream effectors such as adenylyl cyclase and phospholipase C. Subsequent to the receptor and effector coupling, activated GPCRs are phosphorylated by G protein-coupled receptor kinases (GRKs) and/or second messenger-dependent protein kinases, such as protein kinases A and C, that promote the recruitment of β-arrestins, the uncoupling of G proteins to effectors, and, finally, the internalization of receptors inside the cells [31]. The internalization of receptors is thought to be a major desensitization mechanism of GPCRs. Recent studies have shown that not only agonists but also antagonists induce internalization of some GPCRs, including the cholecystokinin receptor [40], the endothelin subtype A receptor [41], the vasopressin V_2_ receptor [42], the serotonin 5-HT_2A_ receptor [43], and the A_1_ adenosine receptor [44], although the mechanisms by which antagonists induce receptor internalization remain unknown. In the case of proton-sensing GPR4, internalization of the receptor is induced by proton as an agonist. However, the GPR4 negative modulator characterized at least in the present study did not promote internalization but inhibited proton-induced internalization, as in the cases for the β_2_-adrenergic receptor and the V_1a_-vasopressin receptor, in which agonists stimulated, but antagonists inhibited, internalization of the receptors [25]. Proton-induced G protein-mediated effector activation and receptor internalization further supports the idea that modes of activation and desensitization of the receptor by proton or hydrogen ions are very similar to those of other ligands, such as amines and peptides.

As antagonists or inhibitors of proton-sensing GPCRs, divalent cations, such as Cu^2+^ and Zn^2+^, [4, 5] and lysolipid molecules, such as psychosine, [6, 36] have been used to characterize receptors. These antagonists or inhibitors may interfere with the protonation of histidine on receptor molecules by acidic microenvironments and, thereby, inhibit subsequent conformational changes of the receptor required for coupling to G proteins [4, 7]. However, high concentrations (~10 mM) of divalent cations are toxic for long-term incubation of cells. For lysolipid psychosine as well, high concentrations more than 20 μM are toxic to cells, and only a narrow range of concentrations, 3~10 μM, can be applied to characterize receptors [6]. Furthermore, the specific antagonistic action of psychosine is not always observed [3]. Therefore, specific antagonists or negative modulators for proton-sensing GPCRs have been anticipated for a long time. The imidazopyridine compounds characterized in the present study showed specificity for GPR4 among proton-sensing GPCRs and can be used to characterize acidic pH-induced actions, at least *in vitro*. Thus, we found that GPR4 can be internalized inside cells in response to an acidic microenvironment, as in the cases of amine and peptide ligand-mediated internalization, in a manner susceptible to compound 1. Recent studies of Yang’s group have shown that GPR4 and cAMP signaling mediates the monocyte adhesion and several proinflammatory gene expression, including chemokines, cytokines, NF-κB pathway genes, and COX-2, in endothelial cells [21, 22]. They also showed that a different imidazopyridine compound from ours inhibited the expression of these proinflammatory genes [22]. We have also shown that compound 1, another imidazopyridine compound, inhibited acidic pH-induced GPR4-mediated proinflammatory adhesion molecule expression in endothelial cells *in vitro*. It should be noted, however, that imidazopyridine compounds employed in the present study were much effective at weak acidic pH or neutral pH but less effective at pH lower than 6.8, implying that we may not expect much improvement of inflammatory disorders, such as atherosclerosis and cancers, where pH is known to reach up to 6.0 [2,12], by *in vivo* treatment of the compounds. Nevertheless, the GPR4 negative modulators can be applied for *in vivo* studies. Indeed, a patent study [23] showed that the imidazopyridine compound prevents neutrophil accumulation and TNF-α production in bronchoalveolar lavage fluids in lipopolysaccharide-induced acute lung injury mouse models. Investigation of the role of GPR4 in inflammatory processes using imidazopyridine compounds is an important subject of our future study.

In conclusion, we characterized some imidazopyridine compounds as GPR4 negative allosteric modulators and compared their action modes with those of psychosine, a selective proton-sensing GPCR antagonist. The GPR4 negative modulator is specific to GPR4 among proton-sensing GPCRs and can be applied to characterize GPR4-mediated biological actions induced by extracellular acidification.

## Supporting Information

S1 Fig Either Compound 1 or psychosine inhibits acidic pH-induced SRE activation in H79F GPR4 mutant. HEK293 cells were transiently transfected with plasmid of wild-type GOR4 or GPR4 mutant (H79F) together with pRL-TK and pSRE-luc. The cells were then incubated for 6 h at the indicated pH to measure SRE promoter activity in the presence or absence of compound 1 (C1; 100 nM) or psychosine (Psy; 10 μM). The activity in GPR4 mutant-expressing cells was expressed as percentages of the value obtained at pH 6.8 in wild-type GPR4. Results are means ± SEM of 4 determinations from two separate experiments. The effect of both test compounds was significantly different from control (*p < 0.05).
(TIFF)
